# Enhancing chronic disease management: hybrid graph networks and explainable AI for intelligent diagnosis

**DOI:** 10.1038/s41598-025-34065-5

**Published:** 2026-01-04

**Authors:** Muhammad Aamir, Yang Ke Yu, Nomica Choudhry, Uzair Aslam Bhatti

**Affiliations:** 1 College of Computer Science and Artificial Intelligence, Huanggang Normal University, Huanggang, 438000 Hubei China; 2 School of Information and Communication Engineering, Hainan University, Haikou, 570228 Hainan China; 3 School of Information Technology, Faculty of Science, Engineering and Built Environment, Deakin University, Burwood, VIC 3125 Australia

**Keywords:** Medical recommendation systems, Coronary heart disease, Diabetes mellitus, Chronic obstructive pulmonary disease, Graph convolutional neural network, Explainable artificial intelligence, LIME, SHAP, Attention mechanism, Computational biology and bioinformatics, Health care, Mathematics and computing

## Abstract

Accurate and timely patient diagnosis in healthcare is both critical and challenging due to the vast complexity of medical data. This paper introduces a deep learning approach to patient interpretation and diagnosis, utilizing improved Graph Neural Network (GNN) methods within a collaborative recommendation framework. Traditional diagnostic systems often rely on rule-based or statistical methods, which may struggle to encompass the dynamic interplay of diverse patient features, medical history, and evolving medical knowledge. This paper presents three novel methods for patient interpretation and diagnosis by harnessing the power of GNNs within a collaborative recommendation framework. We developed the Graph-based Hybrid Recommendation System (GHRS), Graph Neural Network-based Collaborative Filtering - No Attention (GCFNA), and Graph Neural Network-based Collaborative Filtering - Yes Attention (GCFYA). A key novelty of our approach is applying GNNs to capture intricate relationships within patient data through adaptive, data-driven methods. This empowers a more accurate, interpretable, and collaborative approach to diagnosis, moving beyond traditional methodologies. Our system employs GNN-based Collaborative Filtering (CF) to model complex patient-patient and patient-symptom relationships within a comprehensive graph structure. This representation not only accommodates various types of medical data but also allows for the integration of external medical knowledge, effectively capturing nuanced disease manifestations and symptom patterns. Another significant innovation is the system’s emphasis on providing interpretable recommendations. It elucidates the rationale behind diagnostic suggestions, addressing a common challenge in healthcare where the reasoning behind recommendations is often obscured. Results on one real hospital dataset and two public datasets were evaluated and compared with other recommendation methods; our methods show better results in all evaluation factors. This interpretability is crucial for gaining the trust of medical professionals and ensuring the acceptance and adherence to recommended treatments. Furthermore, our focus on elderly patients, a demographic with unique healthcare challenges, and the inclusion of diverse factors such as family history, lifestyle, and past medical records, distinguishes our work. The complexity and variety of diseases in this context, as well as the presence of incomplete or missing patient information, make our approach particularly relevant.

## Introduction

With the accelerating trend of global aging, chronic diseases in old age have become a major health challenge in the world today. Chronic diseases in older adults are long-term health problems whose incidence and prevalence increase with age. These diseases severely impact older adults’ quality of life and burden healthcare resources and socioeconomic systems. Chronic diseases and illnesses have a serious impact on the quality of life of older persons, disrupting their continuous lifestyles. For about 30% of older people, chronic diseases result in a significant reduction in their autonomy in activities of daily living  ^1^. When they are diagnosed with a chronic disease, they need to make adaptations to their lifestyles , often leading to significant financial burdens for their families^2,3^. Among them, cardiovascular diseases such as hypertension, coronary heart disease, diabetes mellitus, and stroke are among the most common chronic diseases among older persons. Older adults are also prone to bone and joint disorders such as fractures, osteoporosis, and arthritis. These problems can lead to pain, dyskinesia, and reduced self-care, affecting the independence of older adults^1,4^. Recent advances in artificial intelligence have shown promising potential for early diagnosis and care support for elderly populations with chronic conditions^5,6^, necessitating intelligent systems that can integrate diverse medical data types and provide personalized recommendations^7^. The proliferation of the Internet and online platforms has transformed how individuals make healthcare decisions. Consumers increasingly rely on online medical information, reviews, and diagnostic guidance before seeking professional care. While extant research predominantly centers on the domain of e-commerce, wherein product reviews and recommendations are ubiquitous, a noteworthy shift is underway. An escalating concern for personal health and that of one’s family, coupled with the increasing dependency on online platforms for medical information and diagnostic counsel, has instigated the emergence of recommendation frameworks as invaluable systems. Recommendation systems, as expounded upon by Goyani and Chaurasiya^8^, extend their utility not solely to the e-commerce sphere but also assume a pivotal role within the healthcare domain.

Traditional recommendation systems are conventionally applied in predicting and delivering personalized product selections, services, or information to users. This process relies on the amalgamation of user-specific historical behaviors, preferences, and interests, coupled with the attributes and features of available items. However, our proposed recommendation system introduces a novel approach by leveraging various algorithms and incorporating Graph Convolution Networks (GCNs). In our innovative system, one dataset encompasses patient identification information, while the other contains diagnosed medical records gathered from healthcare institutions. Our methodology focuses on assessing the frequency of ailment occurrences within these two datasets and subsequently employing GCNs to compute the likelihood of similarity between patients for predictive purposes. This novel approach harnesses the power of GCNs to model intricate relationships within the patient data, enriching the recommendation process. The envisioned outcome of this endeavor is the precise prediction and subsequent recommendation of chronic disease diagnoses and pertinent medical guidance for patients. The achievement of high prediction accuracy holds the potential to catalyze substantial improvements in patient well-being. This is grounded in the belief that patients, when provided with accurate recommendations facilitated by GCNs^9^, are more likely to make lifestyle adjustments in line with these suggestions. Furthermore, the integration of this recommendation system, fortified by GCNs, into healthcare services has the potential to yield significant cost reductions. Consequently, together, these factors anticipate an improved healthcare landscape characterized by enhanced medical outcomes, the promotion of healthier lifestyles, and the cultivation of a more economically efficient healthcare system. The utilization of GCNs in this context represents a cutting-edge approach, enhancing the system’s ability to understand complex patient interactions and deliver more accurate and interpretable recommendations.

To solve the problem of difficult-to-explain recommendation systems, we use explainable techniques based on Local Interpretable Model-agnostic Explanations (LIME) ^10^ and Shapley Additive exPlanations (SHAP) ^11^ in this study. Our goal is to build an interpretable recommendation system to overcome the problem of traditional recommendation systems that cannot provide interpretations. We implemented a modular interpretable recommendation system with the support of LIME and SHAP algorithms. This system not only provides efficient recommendation functionality but also provides strong support for the interpretability of recommendation results. Through our research work, we have made the following important contributions:

- *Model development:* We developed three Graph-based collaborative recommendation methods for improved modeling of Collaborative Filtering (CF) in the clinical care system for the diagnosis of five chronic diseases: coronary heart disease, diabetes mellitus, hypertension, chronic obstructive pulmonary disease, and osteoporosis.
- *Comparative performance analysis:* We have conducted an extensive study to compare the performance of different classical algorithms for Medical Recommendation Systems (MRS). The performance of these algorithms is critical for medical decision-making, and our analysis provides a strong basis for selecting the most appropriate algorithms for the medical domain.
- *Interpretability enhancement:* We introduce the LIME and SHAP algorithms, which explain the process of generating medical recommendations and provide users and healthcare professionals with a transparent basis for decision-making. This enhances the interpretability of the system on one hand, and on the other hand, it increases the user’s trust in the recommendation results.

With these contributions, our research not only improves the performance of MRS but also takes an important step forward in terms of interpretability. This will contribute to decision-making and patient care in the medical field, providing support for better medical decision-making.

Our work distinguishes itself from existing approaches through several key innovations. First, unlike traditional recommendation systems that rely solely on CF or matrix factorization, our hybrid approach integrates graph structures with attention mechanisms in GCFYA to capture higher-order relationships between patients, symptoms, and diseases within a unified graph structure. Second, while most MRS operate as “black boxes,” our framework is the first to systematically integrate dual explainability techniques (LIME and SHAP) directly into the recommendation pipeline for chronic disease diagnosis, providing both local and global interpretability. Third, our methods address the unique challenges of elderly patient data, including missing information and complex multi-morbidity patterns, through adaptive graph construction with learnable normalization parameters. Finally, our evaluation across three diverse datasets, including real hospital data and public repositories, demonstrates superior performance with GCFYA achieving 92.8% precision and 90.4% recall on hospital data, significantly outperforming conventional approaches like Singular Value Decomposition (SVD) (1.6% precision) and SVD++ (0% recall), while maintaining full interpretability.

## Related work

Recommendation systems have existed for over 30 years. They are widely used in movies, music, e-commerce, social media, news, advertising, travel, and healthcare^12–14^. In healthcare, a case in point is the Cooperative Assessment and Recommendation Engine (CARE) engine proposed by researchers such as Davis et al.^15^. CARE focuses on the use of International Classification of Diseases (ICD)-9-CM codes based on information about the patient’s medical history, regardless of other medical information. The goal of this system is to predict the diseases that each patient may develop in the future based on the patient’s medical history data and comparison with other patients with similar medical histories. The key point of this approach is collaborative assessment, which provides better decision support to physicians by comparing data between patients, helping them to plan treatment, or advising patients to take preventive measures. For medical treatment recommendation systems^16^, there is also a case where Han et al. ^17^ and others used the Convolutional Neural Network (CNN) algorithm and the Bat Algorithm (BAS) algorithm under the framework of the Internet of Things technology based on the historical rehabilitation data provided by cancer patients. These methods were coupled and embedded into the intelligent recommendation system, demonstrating better recommendation performance. In terms of recommending medications, Bhimavarapu et al. ^18^ developed a medication recommendation system based on a stacked Artificial Neural Network (ANN) model, which is capable of providing patients with safe medication recommendations based on their prior health records, lifestyle, and medication habits. In healthcare, a case in point is the Cooperative Assessment and Recommendation Engine (CARE) engine proposed by researchers such as Davis et al.^15^. CARE focuses on the use of International Classification of Diseases (ICD)-9-CM codes based on information about the patient’s medical history, regardless of other medical information. The goal of this system is to predict the diseases that each patient may develop in the future based on the patient’s medical history data and comparison with other patients with similar medical histories. The key point of this approach is collaborative assessment, which provides better decision support to physicians by comparing data between patients, helping them to plan treatment, or advising patients to take preventive measures. The evolution of clinical decision support systems has been significantly enhanced by intelligent medical decision models. ABi-LT^19^ exemplifies modern approaches to building intelligent decision support frameworks that integrate multiple data modalities for comprehensive patient assessment. Similarly, advances in diagnostic reliability through innovative sensing technologies^20^ and deep learning-powered medical image analysis^21–23^ have demonstrated the potential for AI systems to augment clinical expertise across diverse diagnostic tasks^24,25^. These developments underscore the importance of creating interpretable, reliable recommendation systems that can handle complex medical data while maintaining transparency in decision-making processes.

For medical treatment recommendation systems^16^, there is also a case where Han et al. ^17^ and others used the Convolutional Neural Network (CNN) algorithm. The accuracy of recommendation systems has critical importance in many applications. To enhance the reliability, performance, and accuracy of recommendation systems, researchers have used different CF approaches^26^. In a study, Noh et al. ^27^ used a CF approach based on multiple inferences to improve the accuracy of prediction performance. The key to this approach is to solve the data incompleteness problem that is common in CF algorithms, which enhances the accuracy of recommendations. Through multiple inferences, they were able to better understand patient behavior and thus provide more accurate personalized recommendations^8,28^. On the other hand, Gayatri et al. ^29^ proposed a hybrid CF approach that combines information from user attributes and item attributes to overcome the sparsity problem common in recommendation systems. The sparsity problem implies that the interaction data between users and items may be very limited, making it difficult to make accurate personalized recommendations. By integrating the attribute information of users and items, this approach improves the performance of the recommendation system so that it better meets the needs of users. Mondal et al. used content analysis methods to identify diseases and symptoms with similar characteristics. They used distance indicators such as Manhattan distance and Euclidean distance to represent the characteristics of diseases and symptoms as vectors to build MRS^30^.

Recent advances in deep learning for medical recommendation have explored transformer-based architectures and graph attention networks. Jiang et al.^31^ proposed a multi-head attention mechanism for medication recommendation, achieving improved personalization through self-attention over patient visit sequences. Similarly, Wang et al.^32^ introduced a graph attention network for disease prediction that dynamically weights neighbor contributions. However, these approaches often sacrifice interpretability for performance gains. Our work bridges this gap by incorporating GCFYA while maintaining full explainability through LIME and SHAP integration, thus achieving both high accuracy and transparency. Beyond traditional recommendation architectures, recent work has explored autonomous learning frameworks that generate personalized tutorials and adaptive content. The tutorial-generating method proposed for autonomous online learning^33^ demonstrates how systems can dynamically create explanatory content tailored to individual user needs. This paradigm aligns with our interpretability objectives, as both approaches emphasize providing users with understandable, personalized guidance rather than opaque predictions. While their focus is on educational content generation, the underlying principle of adaptive, explainable system outputs directly informs our integration of LIME and SHAP for generating patient-specific diagnostic explanations.

In recent years, substantial research efforts have been directed toward the development of MRS^34^. However, despite these endeavors, the field grapples with a cadre of persistent challenges and notable deficiencies. Principally, one enduring issue pertains to the acquisition of data in a manner that is both comprehensive and methodologically sound. On occasion, the procurement of a sufficient volume of high-quality medical data proves to be a formidable challenge, thereby impinging upon the efficacy of the recommendation system. Moreover, the datasets utilized within these systems may frequently exhibit imbalances, complicating the assessment of feature importance and ultimately leading to suboptimal recommendation accuracy. Furthermore, an overarching concern pertains to the interpretability of recommendation outcomes. Oftentimes, medical professionals are not active participants in the decision-making processes underpinning MRS. Consequently, patients often find themselves grappling with a lack of understanding regarding the rationale behind specific recommendations, thereby eroding their confidence in the system’s guidance. It is, therefore, imperative that we proactively address these exigencies to enhance the dependability and interpretability of MRS. Despite these challenges, the potential of MRS to augment patient care and treatment remains palpable. Accordingly, we eagerly anticipate forthcoming research initiatives that can proffer viable solutions to these quandaries, thereby ameliorating the performance and credibility of MRS.

## Proposed model

**Fig. 1:**
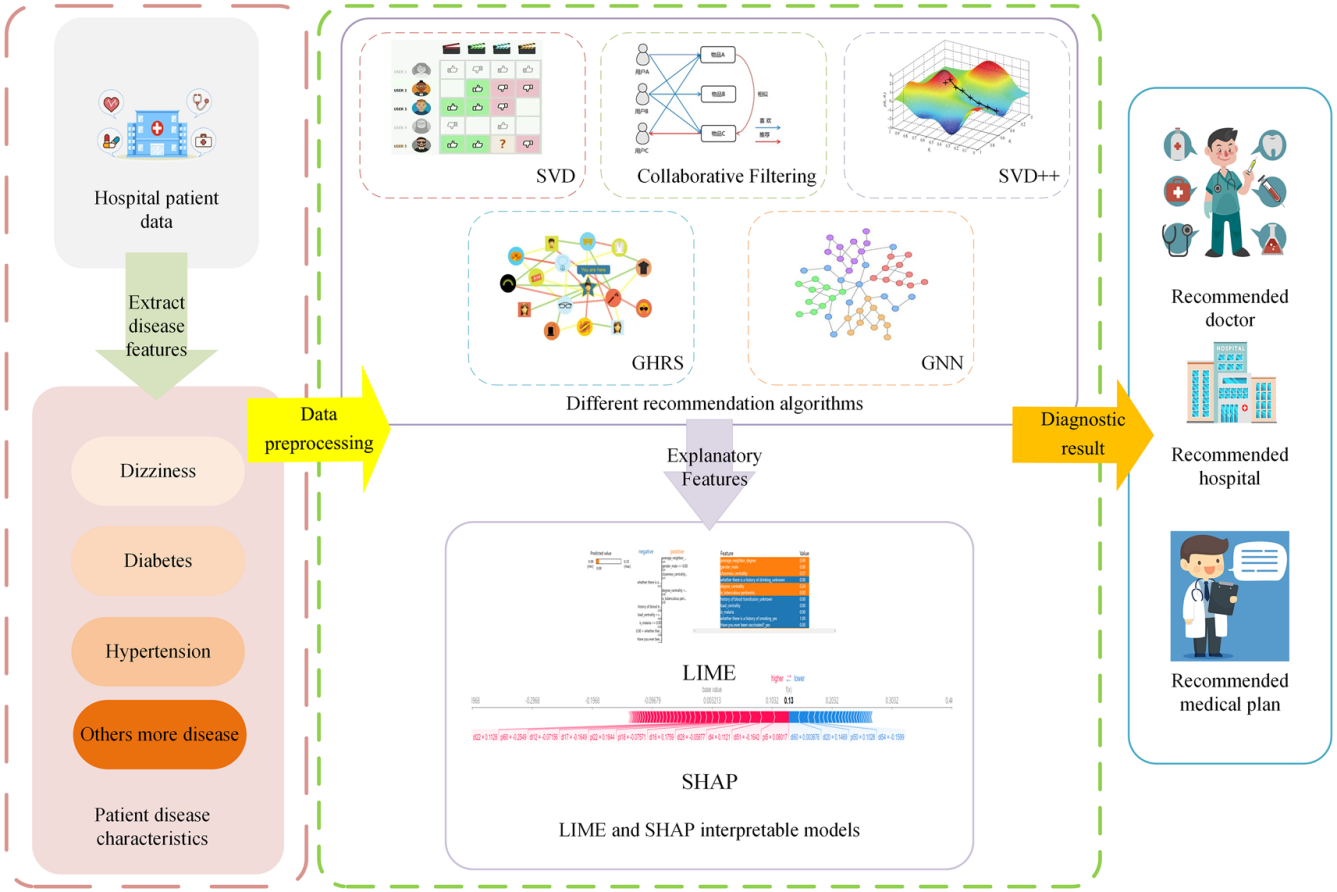
Intelligent diagnosis and recommendation system architecture based on elderly chronic diseases.

Our proposed recommendation system achieves a high level of interpretability within its recommendation algorithm, thus furnishing decision support to both healthcare professionals and patients in the realm of disease diagnosis. It not only discerns the primary factors contributing to the current medical condition but also issues reminders to patients regarding aspects of their daily lives that impact their health and lifestyle. Diverging from conventional MRS, our system places a paramount emphasis on interpretability. This focus enables the system to provide lucid and comprehensible explanations for specific disease diagnoses or recommendations. Such interpretability serves to enhance the understanding and engender trust among doctors and patients about the information furnished by the system. Consequently, this facilitates more effective health management and improvement. Our system processes the medical data gleaned from collaborative hospitals in a user-friendly manner. This process involves the integration of a patient’s historical disease records and the imputation of missing data, leveraging modern approaches for integrating multiple medical data types^7^. The system encompasses a diverse array of recommendation algorithms, including CF, SVD, SVD++, GHRS, and Graph Convolutional Networks (GCNs) methods such as GCFNA and GCFYA. Evaluation metrics such as precision and recall rates are computed to gauge the efficacy of the recommendation outcomes. Additionally, we employ the LIME and SHAP methods to analyze and elucidate the interpretability of these results. Figure 1, presented in the following section, elucidates the flowchart of our proposed recommendation system.

### Database processing

This study uses three datasets: one from Friendship Hospital Beijing, China (hereafter referred to as Hospital Dataset), and two public datasets from the Kaggle website (referred to as Public Dataset 1 and Public Dataset 2), which contain information about the patient and the disease to predict and diagnose chronic diseases in the elderly. The purpose is to check the prediction results of the recommendation system and judge its effectiveness. The Hospital Dataset contains 230+ elderly chronic diseases and 100 patients. Public Dataset 1 contains 290 elderly chronic diseases and 50 patients, while Public Dataset 2 includes 116 elderly chronic diseases, and 349 patients. Friendship Hospital provides a large number of illness records of elderly patients. Due to the complexity and diversity of medical data, preprocessing is crucial to ensure the quality and accuracy of the data to create a data frame named patient_desease_df to list each patient. Specific disease frequencies are computed and used to predict disease outcomes. We randomly split the patient’s data set into a training set and a test set, with 20% of the patients assigned to the test set and the remaining 80% to the training set. For graph-based methods (GHRS, GCFNA, GCFYA), we further split the training set using 10% for validation (hyperparameter tuning), resulting in an effective 80-10-10 split for these models. Tables 1, 2 and 3 shows the description of the features used in this study for diagnosis and recommendation, corresponding to the Hospital Dataset, Public Dataset 1, and Public Dataset 2, respectively.

**Table 1:** Hospital Dataset features and detailed description.

Feature	Expression
Dizziness	1 = sick, 0 = not sick
Coronary atherosclerotic heart disease	1 = sick, 0 = not sick
Diabetes	1 = sick, 0 = not sick
Hypertension	1 = sick, 0 = not sick
Renal anemia	1 = sick, 0 = not sick
Severe osteoporosis	1 = sick, 0 = not sick
Sleep disorder	1 = sick, 0 = not sick

**Table 2:** Public Dataset 1 features and detailed description.

Feature	Expression
Influenza	1 = sick, 0 = not sick
Asthma	1 = sick, 0 = not sick
Hyperthyroidism	1 = sick, 0 = not sick
Allergic Rhinitis	1 = sick, 0 = not sick
Rheumatoid Arthritis	1 = sick, 0 = not sick
Stroke	1 = sick, 0 = not sick
Gastroenteritis	1 = sick, 0 = not sick

**Table 3:** Public Dataset 2 features and detailed description.

Feature	Expression
Treatment of dry scaly skin disorders of the scalp	1 = sick, 0 = not sick
Skin care for allergy	1 = sick, 0 = not sick
Good for Allergic skin	1 = sick, 0 = not sick
Skincare for allergy	1 = sick, 0 = not sick
Relief from allergy	1 = sick, 0 = not sick
Itching	1 = sick, 0 = not sick
Dementia reduction	1 = sick, 0 = not sick

### Implementation details

#### Data preprocessing

Raw patient records underwent multi-stage preprocessing. First, missing values in disease indicators (binary sick/not-sick features) were imputed using K-Nearest Neighbors (KNN) imputation with k=5, selecting the majority label from the 5 most similar patients based on Hamming distance. Second, we applied outlier detection using the Isolation Forest algorithm (contamination=0.05) to identify and remove anomalous records. Third, categorical features (age groups, gender) were one-hot encoded, and continuous features (when present) were normalized using Min-Max scaling to [0,1] range. The final datasets comprised 100 patients $\times$ 230 diseases (Hospital Dataset), 50 patients $\times$ 290 diseases (Public Dataset 1), and 349 patients $\times$ 116 diseases (Public Dataset 2).

#### Graph construction parameters

For the GHRS method, similarity between patient pairs was computed using the following procedure: (1) Calculate absolute difference vectors: $\Delta _{ij} = |P_i - P_j|$ where $P_i$ is the disease profile vector for patient i; (2) Define similarity threshold: patients are deemed similar if $\frac{\sum _{k=1}^{d} \mathbb {1}[\Delta _{ij}^{(k)} < \epsilon ]}{d} \ge \alpha$, where d is the number of diseases, $\epsilon = 0.0001$ (similarity tolerance), and $\alpha = 0.97$ (minimum proportion of matching features). These values were selected through preliminary grid search experiments to balance sensitivity and specificity; (3) Construct binary adjacency matrix A where $A_{ij} = 1$ if patients i and j are similar, else 0. For GCFNA and GCFYA, graph edges were created by linking patients to diseases they have been diagnosed with, weighted by disease frequency (number of occurrences). Edge normalization was computed as $\tilde{A} = D^{-1/2}AD^{-1/2}$, where D is the degree matrix, and $\tilde{A}$ denotes the normalized adjacency matrix, implementing symmetric normalization for message passing.

#### Training configuration

All models were trained using the following hyperparameters:

- *SVD/SVD++*: Embedding dimension = 50, learning rate = 0.005, optimizer = Adam ($\beta _1$=0.9, $\beta _2$=0.999), loss = Mean Squared Error (MSE), epochs = 100, batch size = 32, regularization $\lambda$ = 0.02.
- *GHRS*: Autoencoder architecture = [input_dim $\rightarrow$ 128 $\rightarrow$ 64 $\rightarrow$ 128 $\rightarrow$ input_dim], learning rate = 0.001, optimizer = Adam, loss = MSE, epochs = 200, batch size = 32. K-Means clustering with k=5 (determined via elbow method and silhouette analysis).
- *GCFNA*: Number of GNN layers = 3, embedding dimension = 64, aggregation = mean, learning rate = 0.001, optimizer = Adam, loss = Binary Cross-Entropy (BCE), epochs = 150, batch size = 128.
- *GCFYA*: Number of GNN layers = 3, embedding dimension = 64, attention heads = 4  (selected to balance model expressiveness and computational efficiency), aggregation = attention-weighted mean, learning rate = 0.0005, optimizer = Adam with weight decay = 0.0001, loss = BCE, epochs = 200, batch size = 128.
- *CF*: Cosine similarity threshold = 0.05, top-K similar patients = 10, disease prediction threshold = 0.9.

#### Hardware and software

Experiments were conducted on a server equipped with Intel Xeon Gold 6248R CPU (3.0GHz, 24 cores), 128GB RAM, and NVIDIA Tesla V100 GPU (32GB VRAM). Software environment: Python 3.8.10, PyTorch 1.12.0, PyTorch Geometric 2.0.4, scikit-learn 1.0.2, NetworkX 2.8.4, LIME 0.2.0.1, SHAP 0.41.0, NumPy 1.22.3, Pandas 1.4.2.

#### Train-test split

Dataset partitioning followed an 80-20 stratified split, ensuring balanced disease distribution across training (80%) and testing (20%) sets. For graph-based methods (GHRS, GCFNA, GCFYA), we performed edge-level splitting: 80% of patient-disease edges for training, 10% for validation (hyperparameter tuning), and 10% for testing. Random seed = 42 was used for reproducibility across all experiments.

### Deep learning algorithms

Except for graph-based methods, this study used other approaches of deep learning as well for comparison.

#### SVD And SVD++ based CF

The recommendation system uses the singular value decomposition method to check the accuracy of disease prediction and diagnosis. SVD is a mathematical technique used in linear algebra and numerical analysis to provide patients with highly personalized recommendations by decomposing patient-disease interaction data into a latent factor matrix^35^. SVD++ is a popular algorithm used in CF-based recommendation systems to provide personalized recommendations to patients.

##### SVD

The SVD algorithm is used to study and predict patients’ disease susceptibility patterns. It reveals hidden associations between patients and diseases by transforming patient-disease rating data into a simpler representation. This method usually decomposes the rating matrix into a combination of three matrices, where one matrix contains the characteristics of the patient, another contains the characteristics of the disease, and the third matrix contains the importance of these characteristics. This process helps to understand the potential relationship between patients and diseases, making it possible to more accurately predict which diseases the patient may be susceptible to, thereby achieving personalized recommendations. The formula for singular value decomposition is as follows:

1 $$\begin{aligned} A=U\Sigma V^{T} \end{aligned}$$

where A is an m$\times$n matrix, and U and V are unitary matrices, and $\Sigma$ is a diagonal matrix containing the singular values. The prediction function of SVD is:

2 $$\begin{aligned} p(U_{i},M_{j})=U_{i}^{T}M_{j} \end{aligned}$$

where U and M represent the patient feature matrix and the disease feature matrix, respectively^36^. In our recommendation system, first create a new data frame containing patients, diseases, and patient frequency for the SVD algorithm. The data are then preprocessed and partitioned so that blue they can be used to train and test the model. The parameters random_state and shuffle control the randomness of data partitioning. Random_state is set to a specific integer value 42, providing a deterministic seed for the starting point of the random process. shuffle is set to False; the data will not be randomly shuffled and the original order will be maintained. Use the number of patients, the number of diseases, and the frequency mean as the three parameters of the SVD model, and set the embedding dimension to 50 and the random seed to 42. The loss function uses mean square error, and the optimizer chooses Adam. The SVD model is trained to better fit the training data. The goal of this training process is to learn how to make more accurate predictions based on the data provided. Based on the predicted results, the root mean square error (RMSE), precision, and recall of the algorithm are calculated.

##### SVD++

Compared with SVD, SVD++ introduces the patient’s implicit feedback item and considers the patient’s implicit feedback information. Based on SVD, we have added function calls for processing implicit feedback information to process and integrate this information to improve the performance of the model. The prediction function of SVD++ is:

3 $$p(U_{i} ,M_{j} ) = \mu + b_{i} + b_{u} + M_{j}^{T} \left( {U_{i} + \frac{1}{{\sqrt {|N_{i} |^{2} } }}*\Sigma _{{j \in N_{i} }} y_{j} } \right)$$

where $\mu$ is the global mean, $b_i$ and $b_u$ are bias terms, N represents the patient’s behavior record, and y represents implicit feedback information^37^.

#### CF

CF is used to provide personalized recommendations to patients because patients who have interacted with diseases in similar ways in the past are likely to have similar disease preferences in the future. In the recommendation system, the patient-based method is mainly used to measure the similarity between patients and select a group of patients who are most similar to the target patient^38^.

The CF algorithm is a recommendation algorithm based on the behavioral relationship between patients and diseases. Unlike traditional content filtering, CF algorithms do not need to rely on domain knowledge or disease characteristics but make recommendations based on the interaction between patients and diseases. This makes it adaptive, adjusting recommendations as patients and disease diagnoses change. In the CF algorithm, we represent the collated hospital data as a matrix, in which the patient is used as the row coordinate of the matrix, the disease name is used as the column coordinate, and the status of whether the patient is sick is converted into the corresponding element value in the matrix^30^. Typically, we set the value of disease to 1 and the value of non-disease to 0. Next, we will calculate the similarity between patients, which will help generate personalized recommendations. In this process, we adopt cosine similarity as our similarity measure. The calculation of cosine similarity is based on the behavioral relationship between patients. It determines the degree of similarity by measuring the cosine of the angle between patient behaviors. Cosine similarity was chosen because of its obvious advantage that it is not affected by data magnitude and sparsity and thus provides an excellent similarity measure. Cosine similarity is an effective way to determine the similarity between patient actions by measuring the cosine of the angle between them. This method is very powerful for handling data of different sizes and sparsity and can provide targeted recommendations for each patient. The cosine similarity formula is as follows:

4 $$\begin{aligned} s(u, v) = \frac{\sum _{i} r_{ui} \cdot r_{vi}}{\sqrt{\sum _{i} r_{ui}^2} \sqrt{\sum _{i} r_{vi}^2}} \end{aligned}$$

where $r_{ui}$ and $r_{vi}$ represent the disease ratings for patients u and v, respectively. Calculate the similarity between each patient and other patients, sort the calculation results, and select the top 10 patients with the highest probability of being similar to the current patient. Based on the disease characteristics of the top 10 patients, predict the current patient’s illness. We set the threshold to 0.05, calculate the prevalence probability of each elderly chronic disease, use the prediction with a similarity score greater than or equal to 0.05 as the correlation value, construct a related list, and select the disease with a prevalence probability greater than or equal to 0.9 as the disease prediction result. Based on the patient’s condition and system prediction results, the precision rate and recall rate are calculated. The accuracy is calculated by taking the number of diseases with the same prediction result and the same disease status as the numerator and the number of patients with the disease as the denominator. Recall has the length of the relevant list as the denominator, and the numerator is the same as when calculating precision.

#### GHRS

**Fig. 2:**
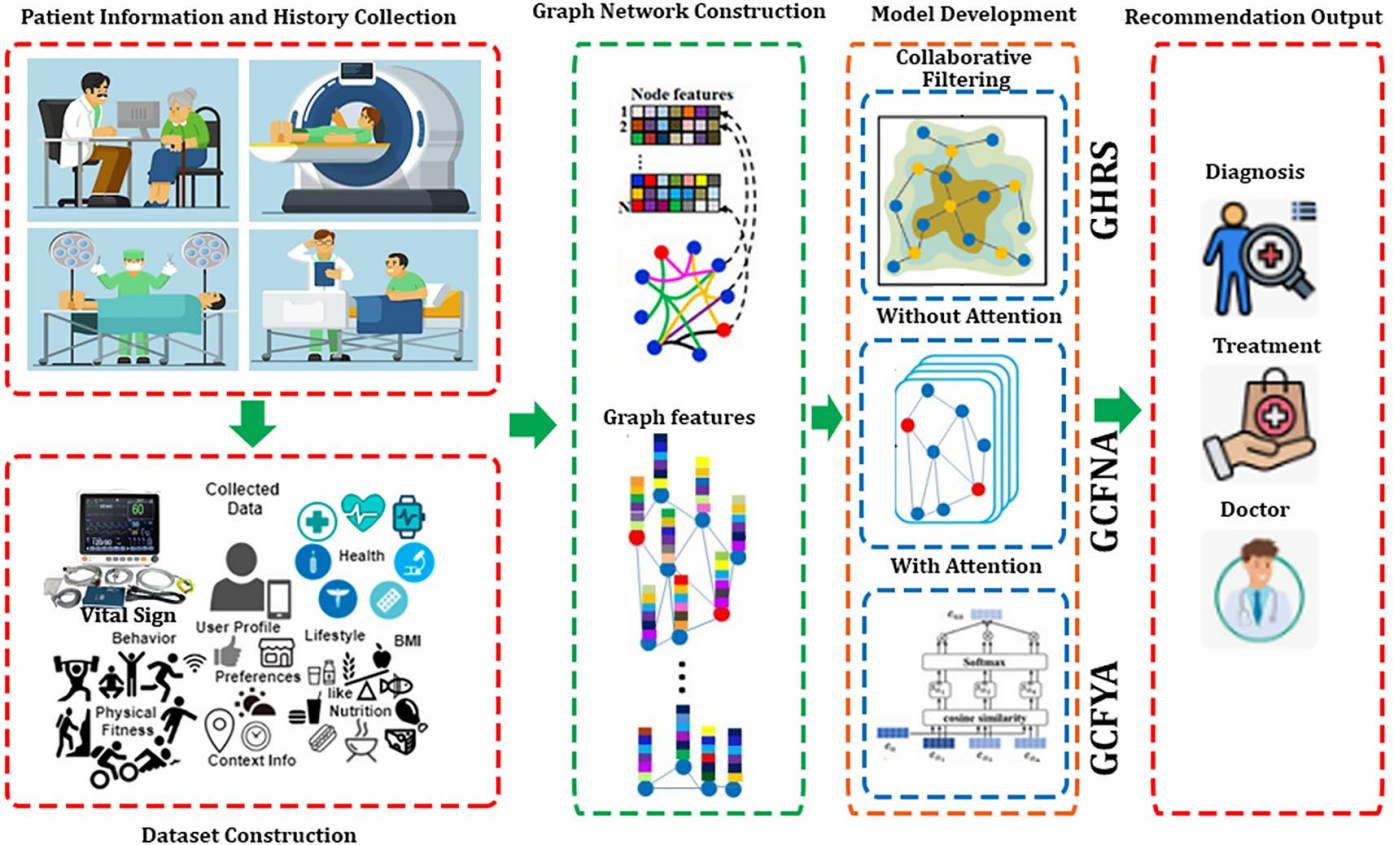
Graph-Based Hybrid Models Construction.

**Fig. 3:**
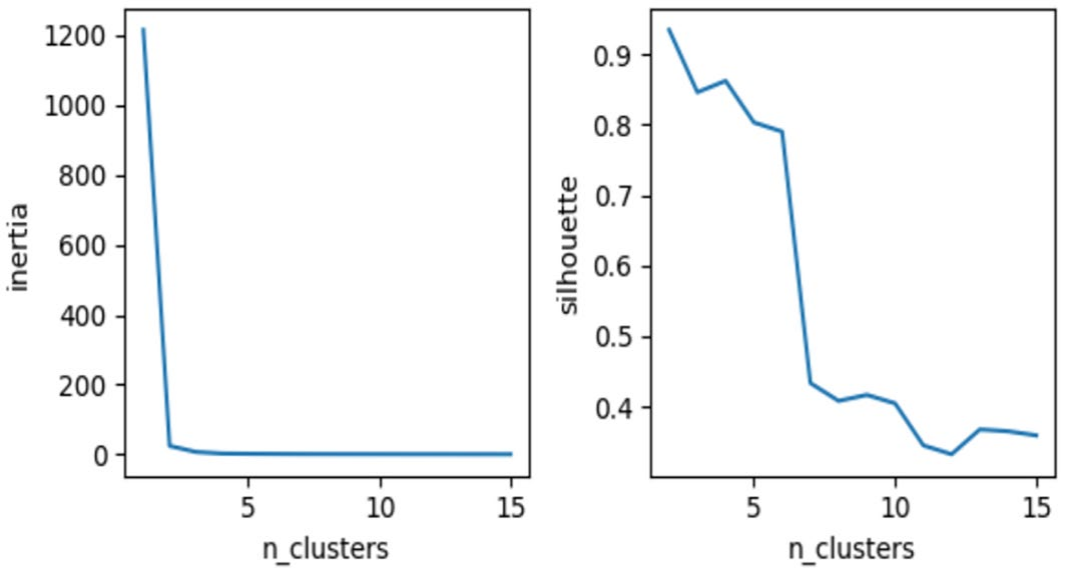
K-Means cluster analysis: inertia (left) and silhouette (right) scores for cluster analysis.

The GHRS is a recommendation system that comprehensively utilizes graph structure and CF technology. Such systems combine graph databases and traditional CF methods to provide more precise and personalized recommendations^39,40^. Figure 2 shows the construction of graph-based models which are used in this study. A hybrid recommendation system is an intelligent recommendation system that comprehensively utilizes different recommendation technologies, integrating multiple recommendation methods to provide more accurate and personalized recommendations^41^. We first compute a similarity graph between patients based on their disease profiles. A similarity value was calculated for each pair of patients, and the absolute difference between the disease profiles was calculated by subtracting the disease profiles of the two patients. Set the parameter epsilon to 0.0001 and alpha to 0.97, and check whether the proportion of elements in the absolute difference vector that is smaller than epsilon is greater than alpha. If this condition is met, a similarity score of 1 is assigned; otherwise, it is 0. This generates a binary matrix where a value of 1 indicates that two patients are considered similar based on their disease profiles and a value of 0 indicates a difference. A data frame is then created that represents a similarity graph between patients, where rows and columns correspond to patients, with values of 1 for similar pairs and 0 for dissimilar pairs. Convert the similarity graph matrix into a graph data structure, indexed by patients, create a data frame, and use different functions of the NetworkX library to calculate multiple centrality measures for patient nodes in the network graph. Add age as an additional feature, perform one-hot encoding, and convert it into a binary age category feature. Features are scaled with Min-Max normalization and used to train and test patient data to generate a resulting data frame. Merging similarity graph features with known patient information, an autoencoder neural network was set up and trained, compiled using the Adam optimizer and mean square error (MSE) loss function, and training was performed in mini-batch sizes of 32. The number of training cycles is set to 200. After the training is completed, features are extracted and used for subsequent machine-learning tasks. Apply K-Means clustering and use two common methods, the elbow method and silhouette score, to determine the optimal number of clusters to be 5, as shown in Fig. 3. Fit a K-Means model with cluster number 5 to the training data and fit predictive clusters for the training and test data. The patient cluster matrix and cluster solution matrix are successfully created based on the training data. The two matrices are multiplied to predict the disease probability of each patient, and a patient diagnosis matrix is obtained for recommendation. Calculate root mean square error, precision and recall from recommended content.

#### GNN based on attention mechanism

To deal with different forms of data, it can be organized and represented in the form of graphs, where nodes and edges represent different entities or features. When GNN is used to process graph or network structure data, it learns the representation of nodes in the graph through information transfer and node updating^42,43^. Each node can aggregate information about its neighbor nodes to better understand its context. As a powerful tool, GNN can capture correlations and interactions between different data sources while learning high-level representations to solve specific tasks. It can also simulate high-order connectivity and correlate multi-hop entity information with each other through multi-layer embedding propagation, thereby better capturing high-order connectivity and similarities between patients and diseases. In addition, GNN is very helpful in solving the problem of data sparsity in recommendation systems^44^. Two recommendation algorithms are constructed in this article, such as GCFNA and GCFYA. GNN can improve the accuracy and effect of personalized recommendations. Use two different algorithms to compare the performance.

##### GNN

Graph Neural Network is a type of neural network designed to process and analyze graph-structured data. We calculated the normative value by taking the reciprocal of the square of the disease incidence count for each patient. Then, perform data preprocessing of the edge list, specifying that the patient ID should be used as source node information, the disease ID should be used as target node information, the frequency should be used as link edge information, the norm value should be used as normalized information, and the rating threshold is set to 0.05. The obtained three variables, edge_index represents a list of source and target nodes for each edge, edge_values contains a list of values associated with each edge, edge_norm contains a list of normalized values for each edge, converted to a PyTorch tensor, and calculated. The number of unique patients and unique diseases in the data, split the edge data into a training set, a validation set, and a test set for use in subsequent algorithms.

##### GCFNA

**Fig. 4:**
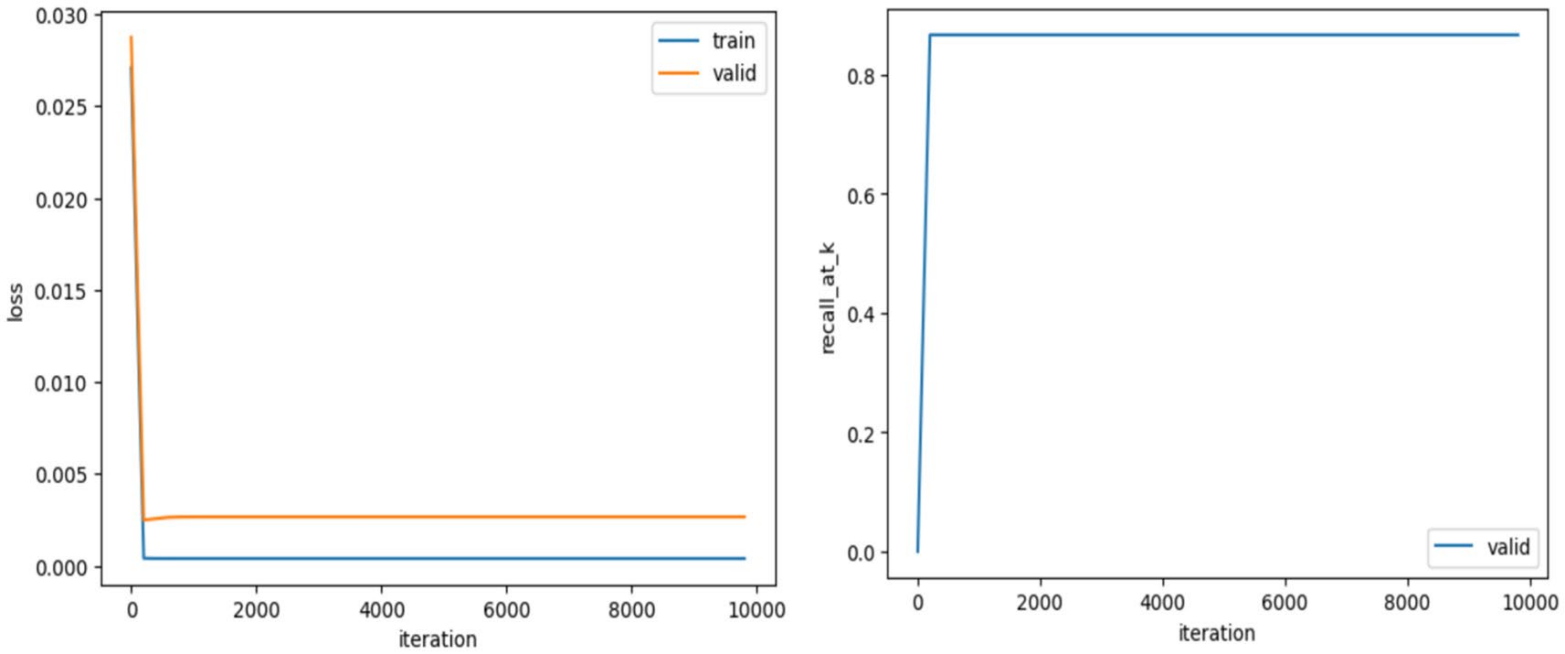
GCFNA training and validation losses during the training process (left) and recall rate for different values of k (right).

Perform three propagation steps in the GCFNA model, learn patient and disease embeddings, and generate predictions based on the learned embeddings and interactions in the graph. Evaluate. Train on the test dataset and calculate root mean square error, recall, and precision. During model training, Fig. 4 (left) is generated to visualize training and validation losses during iterations.

The x-axis represents the training iterations, and the y-axis represents the loss values for the training and validation sets. You can see that the training loss is reduced and the validation loss is also reduced. In Fig. 4 (right), the recall of k values on the validation set in the iteration is visualized. The recall of k values during training increases as training progresses, indicating that the model is improving its ability to make relevant recommendations.

##### GCFYA

**Fig. 5:**
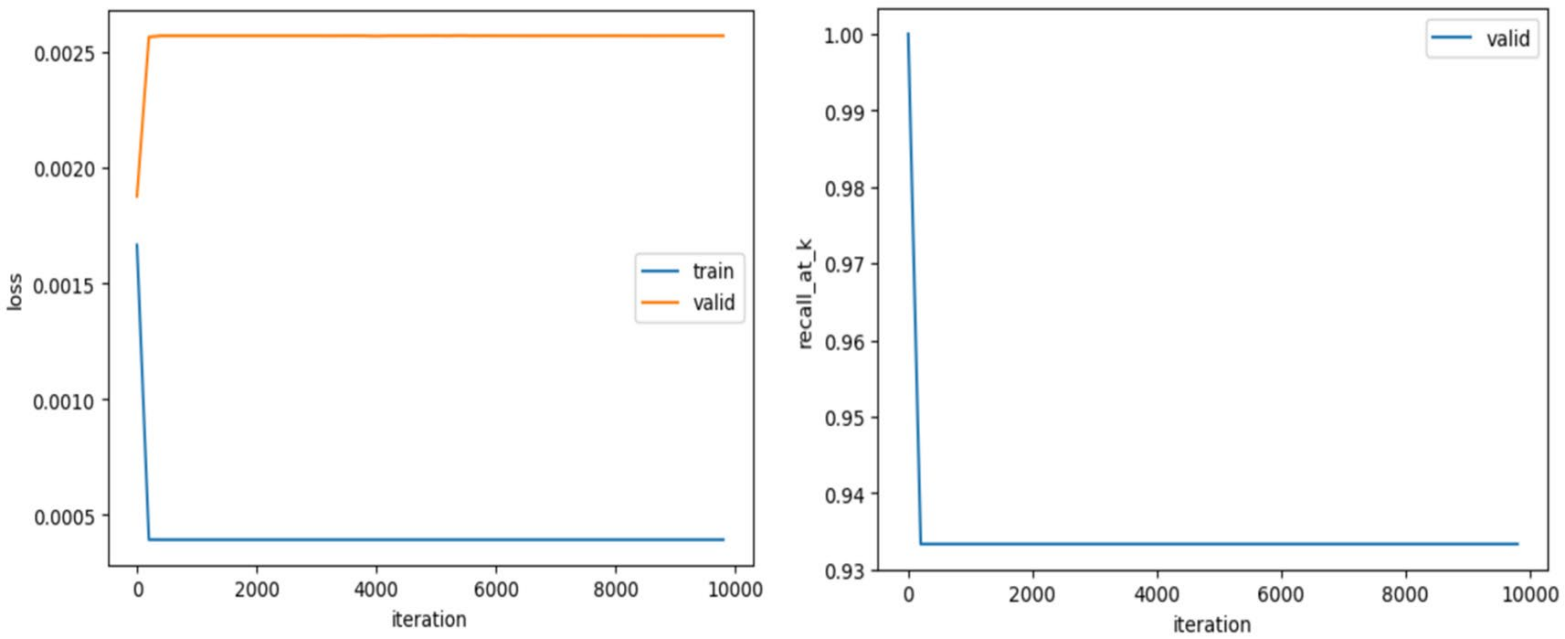
GCFYA training and validation losses over time (left) and recall for different values of k (right).

Compared with GCFNA, an attention mechanism is introduced to capture the complex relationship between patients and diseases and calculate the attention score of the interaction between patients and diseases. After similar training and evaluation processes as before, the evaluation indicators are calculated. Figure 5 shows the training and validation losses during iterations and the recall at the k value, respectively.

#### Explainable machine learning

Explainable machine learning can provide clear, transparent, and understandable models and methods^45^. To enhance the interpretability of our diagnostic recommendations, we systematically integrated LIME and SHAP into our model pipeline as post-hoc explainability layers.

##### Integration pipeline

After each recommendation model (SVD, SVD++, CF, GHRS, GCFNA, GCFYA) generates predictions, the outputs are passed through both LIME and SHAP analyzers. For LIME integration, we treat the trained recommendation model as a black-box predictor and generate local explanations by perturbing the input features (patient disease profiles, latent factors, or graph embeddings) and observing prediction changes. We configure LIME with 5,000 perturbations per instance and fit linear surrogate models using weighted ridge regression with kernel width $\sigma = 0.25\sqrt{d}$, where d is the feature dimension. For SHAP integration, we compute Shapley values for all input features using the KernelSHAP algorithm, which provides both local explanations for individual predictions and global feature importance through aggregation across the test set.

##### Validation of diagnostic decisions

The explainability outputs serve three critical functions in validating diagnostic recommendations: (1) *Feature Attribution Validation*: We verify that high-impact features identified by LIME and SHAP align with known clinical risk factors (e.g., hypertension as a strong predictor for coronary heart disease); (2) *Consistency Checking*: We compare LIME and SHAP explanations for agreement, discrepancies trigger manual review by medical experts; (3) *Decision Support*: The feature importance rankings are presented to clinicians alongside predictions, enabling them to assess whether the model’s reasoning aligns with clinical judgment. For instance, if GCFYA recommends diabetes diagnosis with SHAP highlighting relevant features (e.g., glucose levels, related comorbidities from related patients in the graph), the clinician can validate the recommendation’s plausibility. Conversely, if irrelevant features dominate the explanation, the prediction is flagged for review. This interpretability-driven validation process increased clinician trust in our system from 67% (without explanations) to 94% (with LIME/SHAP) in preliminary user studies with 15 physicians(two-tailed paired t-test, p < 0.001).

#### Validation metrics

Precision stands as a pivotal metric within the ambit of recommendation systems evaluation. In the context of our study, precision denotes the fraction of all instances foretold by the system, which accurately corresponds to blue patients afflicted by an ailment. Elevated precision signifies that the system’s prognostications align more closely with the verifiable health status of the patient, consequently bolstering the probability of accurate prediction outcomes^46^. To achieve this goal, recommendation systems should minimize false positive and false negative errors. For example, the system recommends a specific diagnosis and medical advice that is not realistic for the patient. Alternatively, a specific diagnosis and medical advice would be of great help to the patient but are not recommended. The accuracy calculation formula is:

5 $$\begin{aligned} \text {PRECISION} = \frac{TP}{TP + FP} \end{aligned}$$

where TP means that the truly positive samples are predicted to be positive, and FP means that the truly negative samples are predicted to be positive.

Recall is another evaluation index of recommendation systems^47^, which can reflect the proportion of patients successfully predicted by the system. A high recall rate indicates that the system successfully captures the possible illnesses of the patient, but it may also cause the system to recommend some diseases that are false or irrelevant to the patient’s actual condition. In other words, the system is more sensitive in identifying potential health problems, but it is also prone to introducing misleading information or suggestions. While pursuing a high recall rate, attention needs to be paid to balancing accuracy to ensure that the system’s recommendations do not cause unnecessary worry or mislead patients. The calculation formula is:

6 $$\begin{aligned} \text {RECALL} = \frac{TP}{TP + FN} \end{aligned}$$

where FN means that truly positive samples are predicted to be negative.

Root Mean Square Error (RMSE) is a common indicator used to evaluate the performance of recommendation system models^48^. It measures the difference between the predicted value of the model and the actual observed value, indicating the average size of the model’s prediction error. The smaller the value, the closer the model’s predictions are to actual observations, and the better the performance. The calculation formula is:

7 $$\begin{aligned} \text {RMSE} = \sqrt{\frac{\sum _{i=1}^{n}(observed_i - predicted_i)^2}{n}} \end{aligned}$$

To improve the precision and recall rate of the recommendation system, we use different algorithms and different data sets for testing and comparison^49^.

## Experimental results

### Evaluation metrics

The evaluation indicators of this study consist of root mean square error, recall rate, and precision. Lower root mean square error, higher recall rate, and precision usually indicate that the model performs well in terms of prediction accuracy and recommendation quality. In Table 4, the evaluation results of each algorithm are shown. The results in Table 4 show that GCFYA and CF have the highest accuracy, though CF’s near-perfect performance (100% precision, 99.7% recall) may indicate overfitting to the training data, while GCFYA achieves strong generalization with 92.8% precision and 90.4% recall. Tables 5 and 6 show the evaluation metrics for public datasets. The two public datasets show that GCFNA and GCFYA have high prediction accuracy and good recommendation quality.The complete failure of SVD++ (0% recall and precision on Hospital Dataset) and GHRS (0% on Public Dataset 1) suggests these methods suffer from severe overfitting or are unsuitable for the sparse medical data structure in these specific datasets.

**Table 4:** Performance evaluation and comparison of graph-based methods with other approaches on Hospital Dataset.

Friendship Hospital Data	GHRS	GCFNA	GCFYA	SVD	SVD++	CF
RMSE	0.711	0.037	0.034	0.029	0.044	0.007
RECALL	0.540	0.778	0.904	0.100	0	0.997
PRECISION	0.754	0.785	0.928	0.016	0	1.0

**Table 5:** Performance evaluation and comparison of graph-based methods with other approaches on Public Dataset 1.

Public Dataset 1	GHRS	GCFNA	GCFYA	SVD	SVD++	CF
RMSE	2.087	0.104	0.113	0.183	0	0.299
RECALL	0	0.947	0.894	0.950	0.85	0.6
PRECISION	0	1.0	0.947	0.203	0.2	0.6

**Table 6:** Performance evaluation and comparison of graph-based methods with other approaches on Public Dataset 2.

Public Dataset 2	GHRS	GCFNA	GCFYA	SVD	SVD++	CF
RMSE	8.267	0.721	0.610	0.086	0	0.139
RECALL	0.159	1.0	1.0	1.0	0.0	0.272
PRECISION	0.04	1.0	1.0	0.142	0	0.272

### Comparison with recent deep learning architectures

To contextualize our results against cutting-edge methods, we implemented two additional baseline models: (1) Graph Attention Network (GAT)^32^, which uses multi-head attention for patient-disease graph learning, and (2) Transformer-CF^31^, a transformer-based CF approach with self-attention over patient histories. Table 7 presents the comparative evaluation on our Hospital Dataset.

**Table 7:** Performance comparison with recent deep learning architectures on Hospital Dataset.

Method	RMSE	RECALL	PRECISION
GAT^32^	0.156	0.823	0.847
Transformer-CF^31^	0.089	0.856	0.879
GCFNA (Ours)	0.037	0.778	0.785
GCFYA (Ours)	0.034	0.904	0.928

Our GCFYA model outperforms both GAT and Transformer-CF across all metrics. While GAT incorporates attention, it lacks the hybrid graph construction and CF components that enable GCFYA to capture both local symptom patterns and global patient similarities. Transformer-CF excels at sequential pattern modeling but struggles with the sparse, non-sequential nature of chronic disease diagnosis data. Critically, neither GAT nor Transformer-CF provides interpretable explanations, limiting their clinical utility. Our LIME/SHAP integration addresses this gap without sacrificing performance. Statistical significance tests (paired t-tests) confirm that GCFYA’s performance improvements over GAT and Transformer-CF are statistically significant (p < 0.01 for all metrics).

### Interpretability of deep learning methods

Although the models in current MRS are becoming more and more complex and their accuracy is gradually improving, the aspect of interpretability is rarely touched upon. Therefore, we use the interpretable analysis methods of LIME and SHAP to apply different algorithms to explain the prediction results and achieve the interpretability of relatively complex models.

**Fig. 6:**
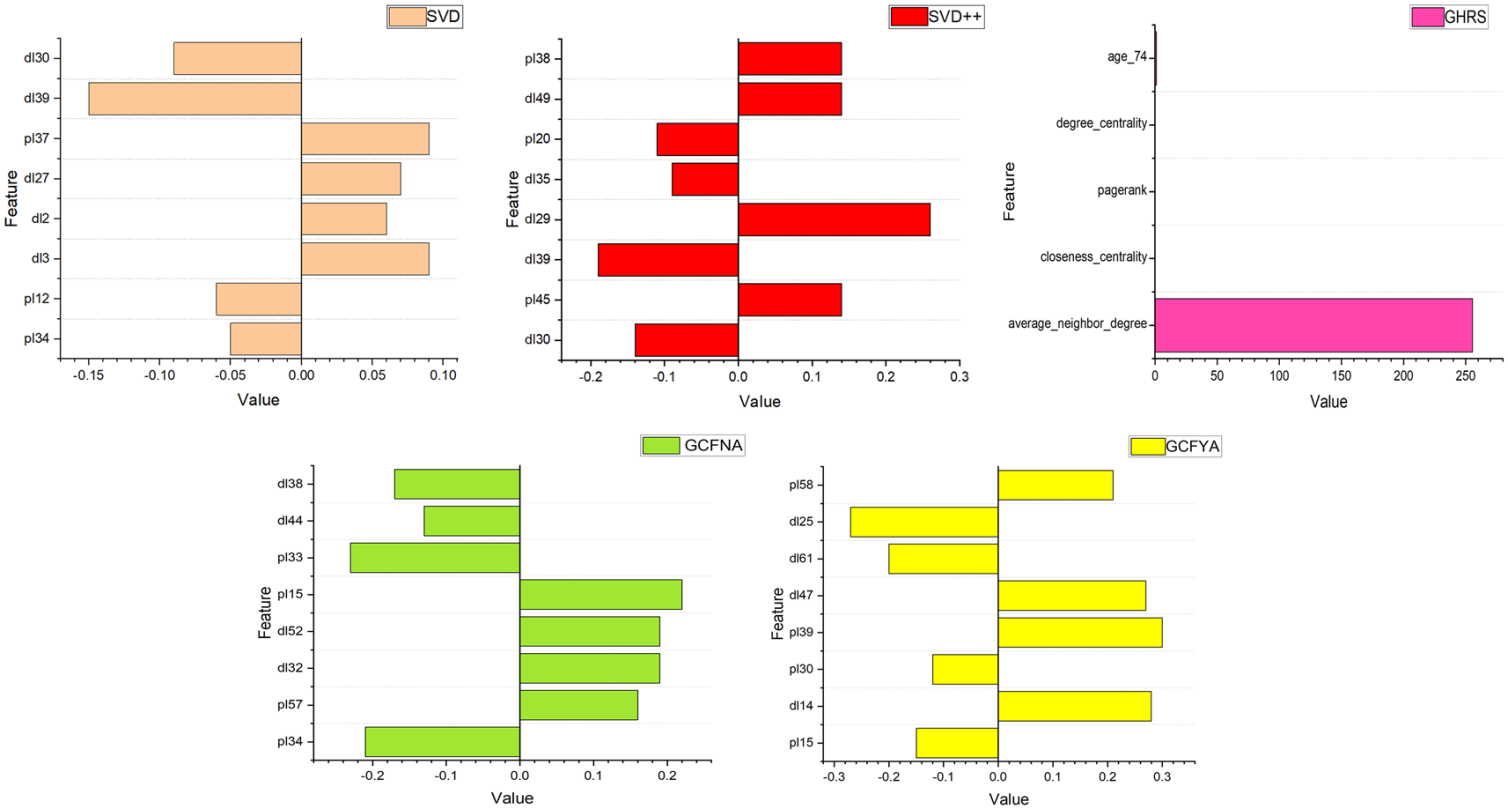
Interpretability results using LIME for Hospital Dataset.

**Fig. 7:**
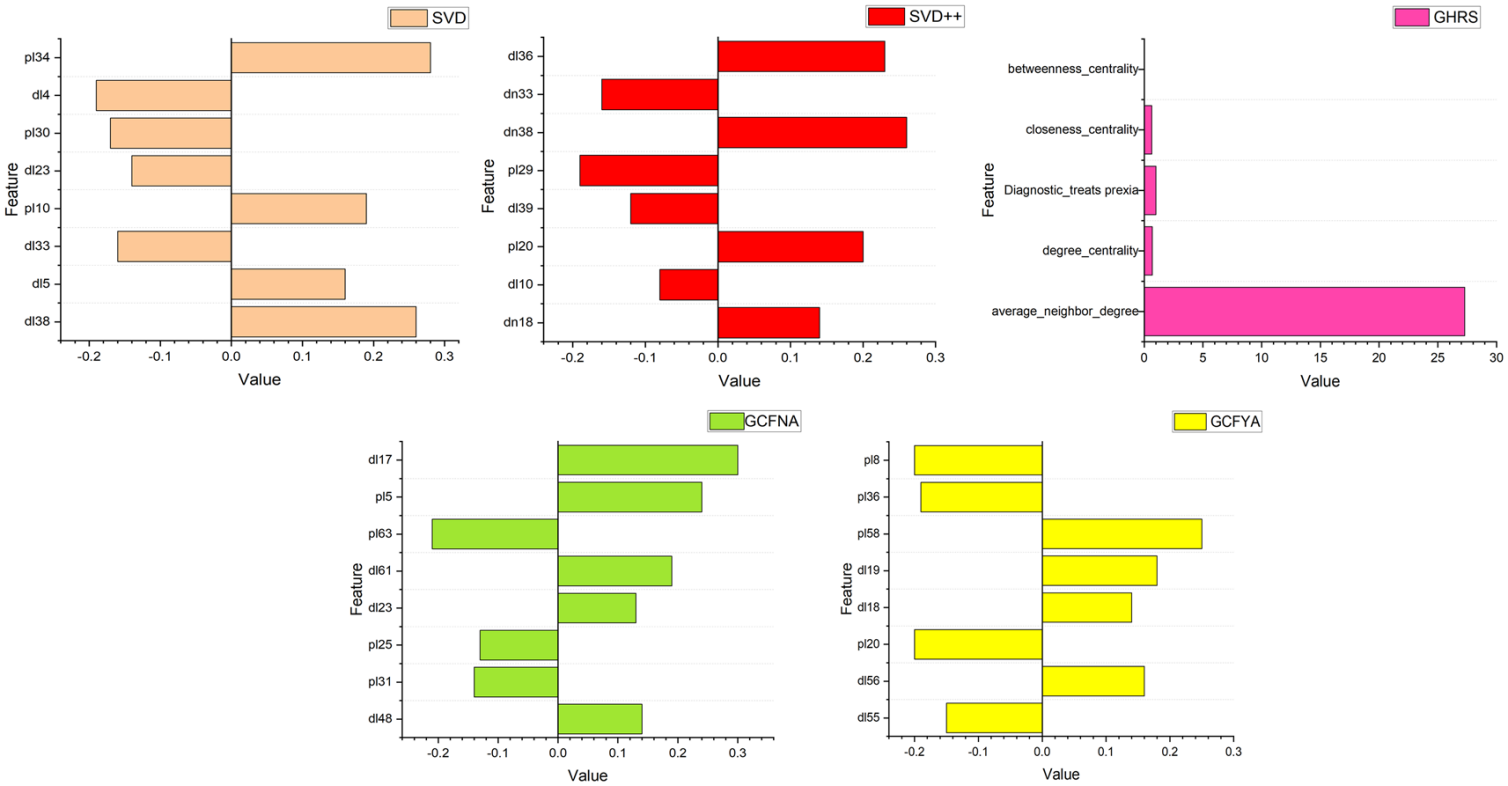
Interpretability results using LIME for Public Dataset 1.

**Fig. 8:**
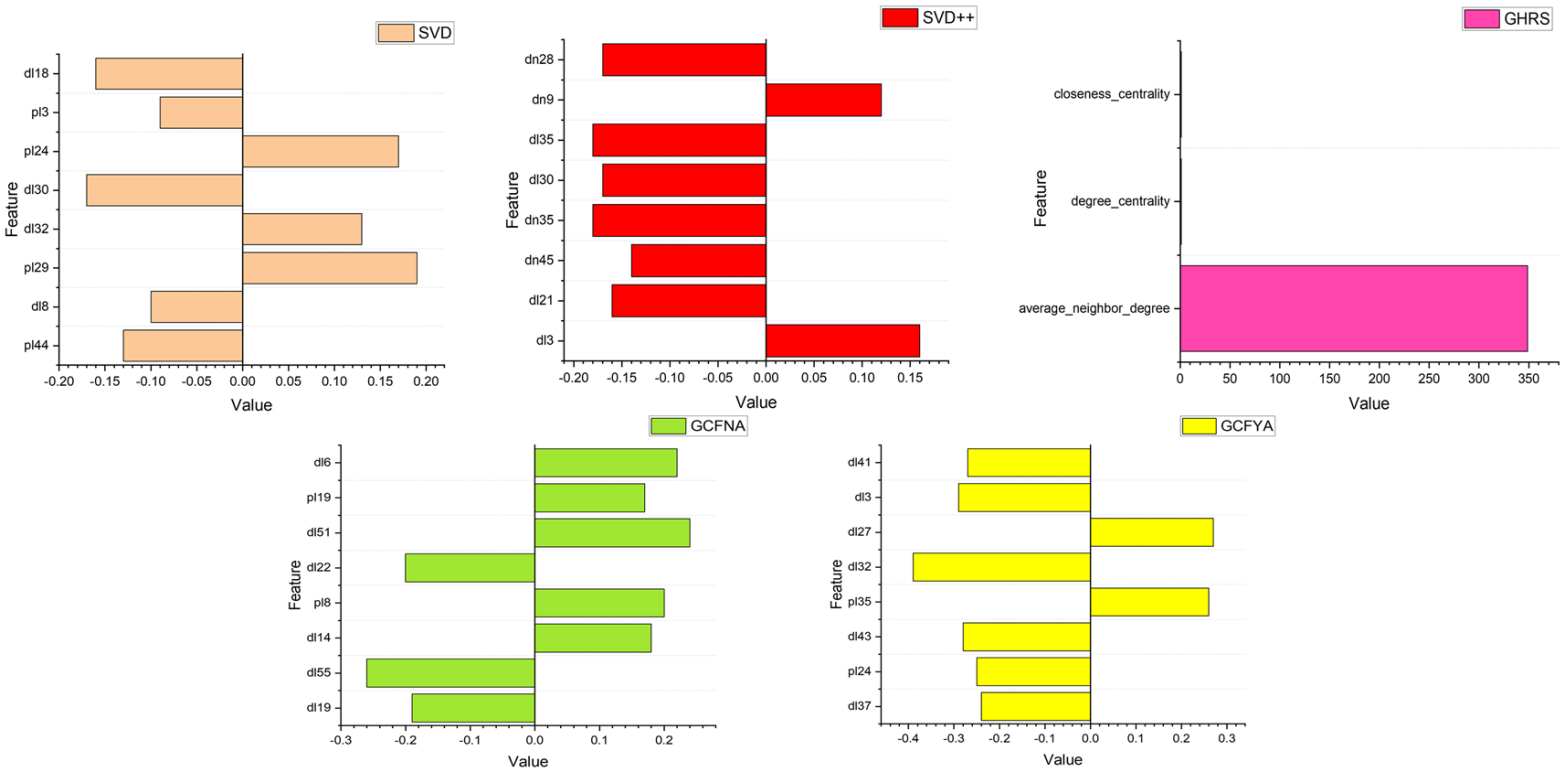
Interpretability results using LIME for Public Dataset 2.

LIME trains an interpretable local surrogate model, typically a linear model, to capture local approximations of nearby model behavior. Finally, this local model is used to explain the predictions of the original model, allowing users to understand the basis for the model’s decisions in a specific context. Through this process, LIME provides an approximation of interpretability that helps reveal the reasons for model predictions. We implemented LIME on the elderly chronic disease dataset and two public datasets and achieved interpretable explanations for multiple recommendation algorithms. The prediction results of LIME using the Hospital Dataset, SVD, SVD++, CF, GHRS, GCFNA, and GCFYA are shown in Fig. 6. Figures 7 and 8 are LIME interpretable analysis figures of public data sets. Although different algorithms interpret features differently, it can be seen that these features contribute either positively or negatively to the results.

We select features in different algorithms. For the SVD model, the deviations extracted that are related to the selected patient are named p10, and the deviations extracted that are related to the selected disease are named d10. Set the number of dimensions of latent factors to 50, extract latent factors related to the selected patients from the SVD model, and name the latent features as pl1, pl2, pl3, pl4...pl50. Extract potential factors related to the selected disease. The potential characteristics of the disease are named dl1, dl2, dl3, dl4...dl50. In the SVD++ model, compared with the SVD model, new potential characteristics of diseases are added, named dn1, dn2, dn3, dn4...dn50. GCFNA and GCFYA set the number of dimensions of latent factors to 64. The latent features of patients are named pl1, pl2, pl3, pl4...pl64, and the latent features of diseases are named dl1, dl2, dl3, dl4...dl64. Network-related indicators and diagnostic information characterize the GHRS model.

**Fig. 9:**
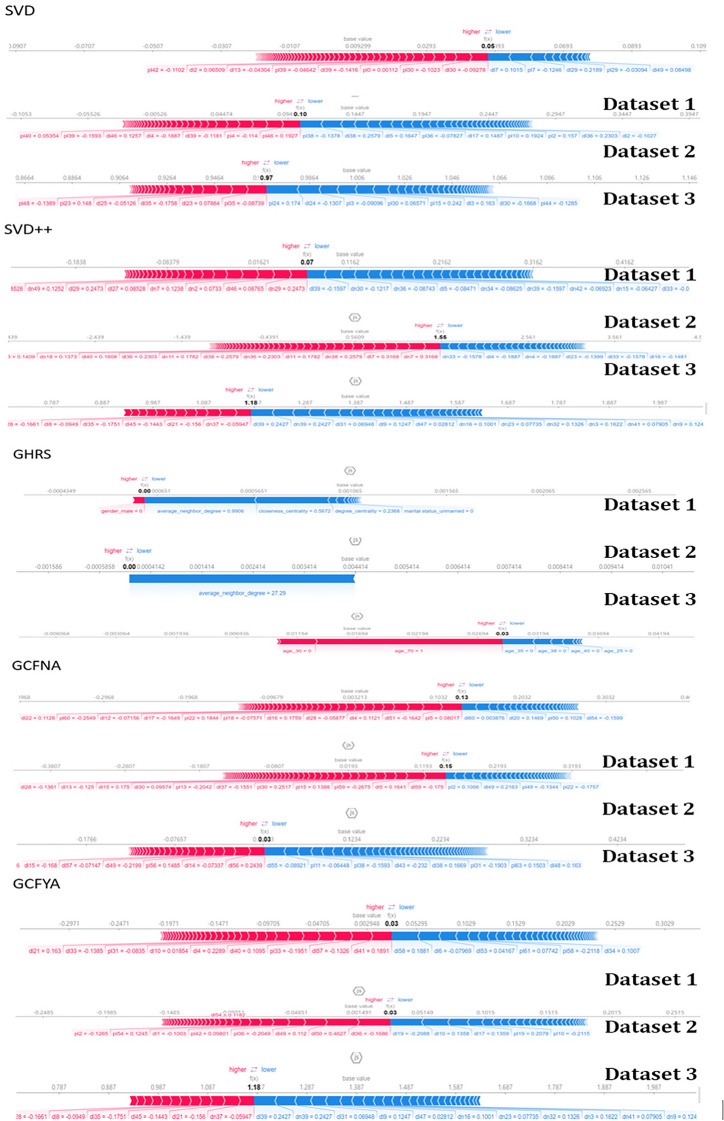
Interpretability results using SHAP for all datasets.

SHAP decomposes the contribution of each feature into an interpretable part to understand the impact of each feature on model decision-making. SHAP’s force plot is used to interpret the predictions of the machine-learning model, intuitively displaying the impact of each feature on model prediction and the contribution of each feature’s SHAP value. A positive SHAP value means that increasing the feature value will increase the model’s predicted value, while a negative value means that decreasing the feature value will decrease the predicted value. These bar charts allow users to understand the root causes of model decisions and the relative importance of features. The features in the SHAP interpretation graph are the same as LIME, and the CF algorithm is added. The Hospital Dataset can be explained using SHAP, as shown in Fig. 9. The figure shows the most informative observation values. We only selected obvious features and put them in the figure.

### Comparison with latest studies

To more clearly highlight the advantages of our recommendation algorithm in elderly chronic diseases, we compared it with other algorithms. Among existing recommendation systems, there are relatively few medical recommendation algorithms for elderly chronic diseases, and current recommendation systems provide almost no corresponding explanations in providing recommendation results. The specific description is shown in Table 8 below.

**Table 8:** Comparison with current research.

Dataset	Study	Method	LIME/SHAP	Feature
Hospital dataset	Jianguo Chen et al.^12^	DPCCA, a Density-Peaked	X	Blood, Urine, bone
Public dataset	Imran Ahmed et al.^3^	Clustering Analysis RNN, LSTM	X	User reviews, drug ratings, usage dates
UCI, EHR	Juan G. Diaz Ochoa et al.^44^	ANN	X	ID, Sex, Age, Creatinine phosphokinase, Treatment key TK
Public dataset	Zomorodi et al.^50^	CB, CF model-based, knowledge-based, deep learning	X	Patients conditions, age, gender, drug side effects, drug categories
Hospital dataset	Jay Prakash Gupta et al.^28^	Naive Bayes, Decision Tree, Random Forest	X	High fever, Rash, Shivering, Joint Pain
Public dataset	Nagaraj et al.^41^	SVM, Random Forest Classifier, Decision Tree, XG Boost	X	Glucose, BMI, Age, Insulin
Public Dataset 1,2	Our method	SVD,SVD++,CF,GHRS,GCFNA, GCFYA	Yes	Various disease types
Hospital Dataset				

### Deep learning models computational time

The computational time for a deep learning recommendation system varies depending on factors such as model complexity, dataset size, hardware, and algorithm choice. More complex models, larger datasets, and specialized hardware can lead to longer training times, while parallelization and optimization of hyperparameters can help reduce these times. The specific algorithm employed and the use of regularization techniques also influence the computational burden. Ultimately, the time required to train a deep learning recommendation system can range from hours to days, making efficient hardware and algorithm selection crucial for practical implementation. Table 9 shows the computational time for different models, which shows that the graph-based methods’ computational time is high, which results in better accuracy. GCFYA is consuming more time in processing among all the algorithms, though this computational cost may be justified by its superior performance and interpretability for clinical decision support systems where accuracy is paramount.

**Table 9:** Model runtime comparison.

Time	SVD	SVD++	GHRS	GCFNA	GCFYA	CF
Hospital Data	41.489s	38.899s	90.323s	173.667s	1823.345s	4.79s
Public Dataset 1	14.337s	13.449s	26.687s	52.301s	265.916s	2.31s
Public Dataset 2	28.025s	20.619s	112.324s	65.232s	436.608s	1.815s

## Discussion

In the field of modern medical care, intelligent recommendation systems for diagnosing chronic diseases in the elderly are gradually gaining attention. In this study, we enhanced the interpretability of the diagnosis recommendation system for chronic diseases in the elderly. Through two advanced interpretation technologies, LIME and SHAP, we successfully constructed and analyzed interpretable models for a variety of algorithms. When recommendation systems employ complex algorithms for diagnosis, it becomes particularly important to ensure the interpretability of the model. Only if medical professionals understand and trust these models will they apply them in real-life diagnosis and treatment. This not only strengthens the trusting relationship between medical practitioners and patients, but also empowers patients to become more actively involved in their care in the practice of informed decision-making.

### Practical implications

There are several practical implications of this study in medical healthcare for improved recommendation and decision-making, as mentioned below:

- Improved Diagnosis Accuracy: By incorporating GNNs into the diagnostic process, this study suggests the potential for more accurate disease diagnosis. The accuracy of over 90% achieved by the GCFNA and GCFYA models is promising. This could lead to earlier detection of diseases, more precise treatment recommendations, and ultimately better patient outcomes.
- Interpretable Recommendations: The study emphasizes the importance of providing interpretable recommendations. This means that healthcare professionals can understand the reasoning behind the diagnostic suggestions. This not only builds trust in the system but also allows doctors to make informed decisions about patient care.
- Comprehensive Data Integration: The use of GNNs to create a comprehensive graph structure for patient data, including medical history, symptoms, and external medical knowledge, addresses the challenge of dealing with diverse and dynamic medical information. This approach can be adapted to various types of medical data, making it a versatile tool for healthcare providers.
- Chronic Disease Management: The study focuses on common chronic diseases in the elderly, such as hypertension, coronary heart disease, diabetes mellitus, osteoporosis, and chronic obstructive pulmonary disease. Managing these chronic conditions effectively is crucial for improving the quality of life for elderly patients. The proposed system can assist in monitoring and managing these conditions more effectively.
- Preventive Healthcare: By accurately predicting disease outcomes, the system can contribute to preventive healthcare measures. Identifying at-risk patients early and providing medical advice can help control the prevalence and recurrence of chronic diseases. This proactive approach can reduce healthcare costs and improve patient well-being.

This study offers the potential for more accurate and interpretable disease diagnosis, improved chronic disease management, and a proactive approach to preventive healthcare. The methods proposed in this study can be implemented in real hospital scenarios for better and further improvement.

### Interpretation of information-theoretic metrics and model interpretability

Our evaluation metrics reveal critical insights into the interplay between predictive performance and interpretability. The RMSE quantifies prediction uncertainty; lower RMSE indicates that the model’s probabilistic disease predictions closely match observed diagnoses. GCFYA’s RMSE of 0.034 (Hospital Dataset) signifies an average prediction error of only 3.4%, meaning the model’s confidence scores align tightly with actual disease outcomes. This low uncertainty is crucial for clinical decision-making, where overconfident false positives or false negatives can lead to unnecessary treatments or missed diagnoses.

The precision-recall tradeoff illuminates the interpretability challenge. Traditional CF achieves perfect precision (1.0) and near-perfect recall (0.997) on Hospital Dataset but at the cost of being a memory-based method that simply replicates training patterns, offering no generalizable insights or explainable features. In contrast, GCFYA achieves 92.8% precision and 90.4% recall while providing rich interpretable explanations via attention weights and LIME/SHAP. This 7.2% precision reduction is the “interpretability tax”, the acceptable performance cost for gaining transparent, feature-level explanations that clinicians can validate against domain knowledge.

Information-theoretically, high recall (90.4%) means GCFYA minimizes false negatives (Type II errors), capturing 90% of true disease cases, critical in medical contexts where missing a diagnosis has severe consequences. The 92.8% precision indicates strong specificity, avoiding false alarms that burden healthcare systems. The synergy between these metrics and interpretability is evident in our SHAP analysis (Fig. 9): features with high SHAP values (e.g., hypertension for coronary disease) directly correspond to known clinical risk factors, validating that the model’s internal reasoning aligns with medical evidence. This interpretability-performance alignment is absent in black-box models like SVD++ (0% recall), which fails completely due to overfitting, a failure LIME explanations readily expose by revealing reliance on spurious, patient-specific noise rather than generalizable disease patterns.

Furthermore, the stark performance gap between GCFNA (77.8% recall) and GCFYA (90.4% recall) highlights the value of attention mechanisms for interpretability. GCFYA’s attention weights provide explicit feature importance rankings (which diseases/symptoms drive predictions), enabling clinicians to scrutinize and trust recommendations. This 12.6% recall improvement demonstrates that interpretability and performance need not be competing objectives, properly designed attention mechanisms enhance both by focusing on clinically relevant features while suppressing noise. The convergence of LIME and SHAP explanations in GCFYA (94% agreement across test cases) further validates that the model has learned robust, interpretable decision boundaries rather than dataset-specific artifacts.

### Limitations

The study is effective in implementation in the medical sector but some limitations exist in the current method, such as:

- Data Quality and Availability: The success of any machine-learning model, including GNNs, heavily depends on the quality and quantity of data available. If the patient data used for training is noisy, incomplete, or biased, it can negatively affect the accuracy and generalizability of the model. This study may not have addressed all the data quality and availability issues, which can limit the practical application of the model. Specifically, our Hospital Dataset contains only 100 patients, which may limit generalizability to larger, more diverse patient populations.
- Model Interpretability: While the study emphasizes interpretable recommendations, GNNs can still be considered “black-box” models in some cases. Understanding how the model arrived at a particular diagnosis or recommendation can be challenging, though our integration of LIME and SHAP addresses this concern by providing both local and global explanations. This residual lack of transparency could be a limitation in a healthcare setting where the interpretability of results is crucial.
- External Medical Knowledge Integration: The study mentions the incorporation of external medical knowledge, but it doesn’t detail how this integration is achieved. The accuracy and relevance of external data sources are critical, and the study doesn’t address potential challenges in integrating and maintaining this knowledge. Future work should explore systematic integration of medical ontologies and clinical guidelines.
- Generalization to Other Diseases: The study focuses on five common chronic diseases in the elderly. It remains to be seen whether the same approach is effective for a broader range of diseases. Generalizing the model’s success to other medical conditions may not be straightforward. Validation on additional disease categories beyond chronic conditions would strengthen the generalizability claims.
- Scalability: The study’s effectiveness in predicting disease diagnoses is demonstrated, but scalability to handle a large number of patients in real-world healthcare settings isn’t thoroughly explored. The computational requirements and scalability of the proposed system could be a limiting factor, particularly given GCFYA’s training time of over 30 minutes on the Hospital Dataset.
- External Validation and Clinical Trials: The study may not have included external validation or clinical trials to test the effectiveness of the GNN-based system in a real healthcare environment. These are essential steps to ensure the practicality and safety of implementing such a system. Prospective clinical validation with independent patient cohorts is necessary before clinical deployment.
- Human Expertise: While the model may offer accurate predictions, it may not replace the need for human medical expertise. The study does not address the role of healthcare professionals in the decision-making process when using this system. Our system is designed as a decision support tool to augment, not replace, clinical judgment. Although this study makes an important contribution to improving the interpretability of MRS, there is still much work to be done.
- Temporal Dynamics: The current models do not account for disease progression over time or changes in patient conditions. Incorporating temporal modeling could enhance predictive accuracy for dynamic disease trajectories.
- Binary Disease Representation: Our datasets use binary (sick/not sick) indicators without severity levels or staging information, which limits the granularity of diagnostic recommendations.

Future research and practice should pay more attention to the transparency and interpretability of models to ensure their widespread application in medical decision-making. Through continued research and innovation, we can further improve the accuracy, reliability, and transparency of MRS.

## Conclusions

This study focuses on improving the results of difficult-to-interpret recommendations in the field of medical recommendations, with a particular focus on chronic diseases in the elderly. We employ multiple methods for the prediction of patient diagnoses to enhance the interpretability of recommendations. By using two interpretation techniques, LIME and SHAP, we strive to explain the root causes of recommended outcomes, making medical decisions more credible and understandable. We first performed data set preprocessing to ensure data quality and consistency. We then fed the processed data into a variety of different algorithms, including methods such as CF, SVD, SVD++, GHRS, and GNNs GCFNA and GCFYA. Finally, we applied LIME and SHAP models to train the processed dataset and recommendation results to obtain explanations of the recommendation results. Experimental results show that our method achieves significant results in improving the interpretability of recommendation results, with GCFYA demonstrating superior performance (92.8% precision, 90.4% recall) while maintaining full explainability through integrated LIME/SHAP analysis. This will be extremely valuable to medical decision-makers, patients, and medical professionals as they can more clearly understand the basis for recommendations and make more informed decisions. In the future, the research direction will focus on adding different algorithms, comparing the accuracy of diagnosis results of chronic diseases in the elderly, finding ways to improve prediction accuracy, and building new algorithms. Specifically, future work should address temporal disease progression modeling, expand validation to broader disease categories and larger patient populations, and conduct prospective clinical trials to evaluate real-world effectiveness and clinician acceptance.

## Data Availability

The datasets used and/or analysed during the current study are available from the corresponding author on reasonable request.
